# Assessment of the Variability of Human Exposure to Radiofrequency Electromagnetic Fields Arising from 5.9 GHz Vehicular Communication in Urban Environments

**DOI:** 10.3390/s23156802

**Published:** 2023-07-30

**Authors:** Gabriella Tognola, Martina Benini, Marta Bonato, Silvia Gallucci, Marta Parazzini

**Affiliations:** 1 Cnr-Istituto di Elettronica e di Ingegneria dell’Informazione e delle Telecomunicazioni (CNR-IEIIT), 20133 Milan, Italy; martina.benini@ieiit.cnr.it (M.B.); marta.bonato@ieiit.cnr.it (M.B.); silvia.gallucci@ieiit.cnr.it (S.G.); marta.parazzini@ieiit.cnr.it (M.P.); 2 Dipartimento di Elettronica, Informazione e Bioingegneria, Politecnico di Milano, 20133 Milan, Italy

**Keywords:** radiofrequency exposure, specific absorption rate, vehicular communication, V2V, field propagation model, urban settings

## Abstract

This paper assessed the variability of radiofrequency exposure among road users in urban settings due to vehicle-to-vehicle (V2V) communication operating at 5.9 GHz. The study evaluated the absorbed dose of radiofrequencies using whole-body specific absorption rate (SAR) in human models spanning different age groups, from children to adults. To overcome limitations of previous studies, we developed a novel hybrid procedure that combines deterministic and stochastic approaches, enabling assessment across multiple urban layouts. Real urban conditions and varying propagation scenarios were considered in SAR calculations. By varying the road user’s position within 1.5–300 m from transmitting cars, the SAR distribution was determined. Median SAR remained consistently low, around 0.70 mW/kg, even with multiple transmitting cars and multiple emitting antennas, using maximum power allowed in US (44.8 dBm). The 99th percentile of SAR distribution varied based on body mass, decreasing for heavier models (typically adults) and increasing with the number of transmitting cars and antennas. The highest absorbed dose (73 mW/kg) occurred in a child model. The SAR consistently remained below the 80 mW/kg limit for whole-body exposure to electromagnetic fields in the 100 kHz–300 GHz range.

## 1. Introduction

The automotive field is constantly evolving thanks to rapid and widespread technological advancements such as those that are leading to the development of connected cars. In modern and future scenarios, connected cars are capable of establishing communication and data exchange with other vehicles, the infrastructure, and pedestrians. This enables them to share real-time traffic information and alert signals, such as in situations involving car accidents, road interruptions, or obstacles. The technologies employed to enable the mentioned service of connected cars produce electromagnetic fields (EMF). Vehicular communication operates through two wireless access technologies, namely, WiFi for mobility (IEEE 802.11p) [1] and cellular technology for vehicle-to-everything communication (C-V2X) [2,3,4,5,6]. In the United States, WiFi-based vehicular communication utilizes the IEEE 802.11p standard. It is commonly referred to as ITS-G5 in the European Cooperative Intelligent Transport Systems (C-ITS) initiative [7]. C-V2X refers to cellular-based V2X communication and is supported by standards such as 3GPP (Third Generation Partnership Project) [5,6]. It enables communication between vehicles (V2V), as well as between vehicles and infrastructure (V2I), vehicles and the network (V2N), or other road users (V2P). Both WiFi for mobility and C-V2X operate within the ITS band at 5.9 GHz [1,7].

The purpose of the current study was to evaluate the variability of the exposure to the radiofrequency (RF) field generated in vehicular communication, with a specific focus on V2V communication operating at 5.9 GHz, and its impact on road users. The current research builds upon our previous studies [8,9,10,11] on RF exposure in V2V 5.9 GHz communication and seeks to provide more generalized and comprehensive results.

**Figure 1:**
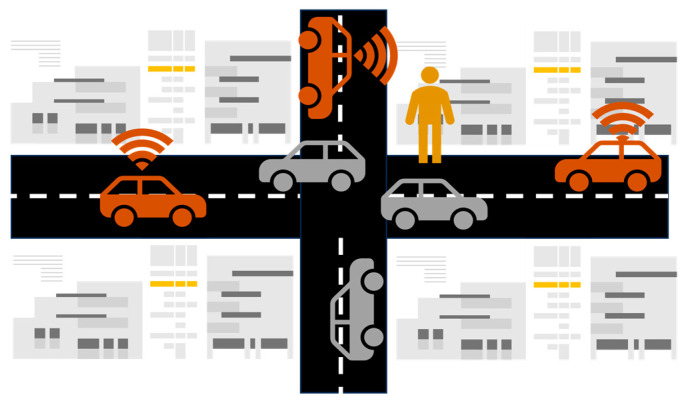
Pictorial representation of the V2V exposure scenario investigated in the current study (please note that the objects displayed in the figure are not in scale). The scenario includes multiple transmitting cars (shown in red) surrounded by non-transmitting vehicles (shown in gray) and a road user (shown in yellow).

In contrast to the previous studies that used simplistic scenarios to assess RF human exposure in vehicular communication [8,9,10,11], the current research takes a more thorough approach. It considers additional factors that reflect real-world conditions of V2V communication, acknowledging the complexity of such environments. Figure 1 illustrates the V2V exposure scenario under investigation. Our objective was to evaluate RF exposure within an urban layout (including buildings and roads) caused by multiple transmitting cars (shown in red) surrounded by non-transmitting vehicles (shown in gray). We assumed that the RF-EMF was emitted by V2V antennas mounted on the roof of the transmitting cars. To calculate the exposure dose, we considered that the road user stood alongside the roads.

In the previous studies [8,9,10,11], the exposure dose was determined in an exposure scenario that comprised a 3D model of a transmitting car equipped with V2V antennas and a human model which was placed either close to the transmitting car to simulate a pedestrian [8,9,10] or inside the car to simulate a passenger [11]. Additionally, in these latter studies, the transmitting car and the human model were placed in the air and the dose absorbed by the human model was obtained by considering only one transmitting car in the scenario with no obstacles or scatterers along the optical path between the transmitter (the car) and the receiver (the road user). 

In contrast, our study incorporated a more realistic approach by introducing several sources of variability. We evaluated exposure scenarios with multiple transmitting cars at random distances from the road user, considering two distinct traffic conditions: normal traffic density and high traffic density. The transmitting cars were placed in realistic urban layouts, taking into account the effects on the electromagnetic field propagation caused by the reflections over the ground and the presence of buildings and other vehicles in the scene, which may act as obstacles and scatterers to the field generated by the transmitting cars. Moreover, we accounted for the variability due to the dimensions of the vehicles, particularly the height of the cars around the transmitting car(s). This aspect significantly influences the way in which the field emitted by the transmitter propagates. To incorporate this variability, we introduced the height of cars as a random variable in our analysis. On the other hand, the results in [8,9,10,11] were obtained for one model of a car with fixed dimensions. Finally, apart from modeling the effects of different urban layouts and field propagation conditions as described earlier, we also sought to account for the variations in exposure resulting from diverse operational conditions of V2V communication and the anatomical characteristics of road users.

Below is a summary of the sources of variability considered in the current study on RF exposure in V2V communication at 5.9 GHz.

In terms of urban layout, our study specifically examines the following factors:

1. The distance between the road user and the transmitting car(s): we aimed to analyze how the distance of the road user from the transmitting vehicle(s) affects the exposure.
2. The propagation conditions along the optical path: this refers to the characteristics of the environment through which the exposure field generated by the V2V antennas passes. It includes factors such as the layout of roads, buildings, and objects in the scene (such as other cars). By using a stochastic approach, we investigated how these elements influence the propagation of the exposure field between the transmitting car(s) and the road user.

Regarding the operating conditions of V2V communication, our study focused on the following aspects:

3. The power level at which the V2V antenna(s) are operated: we investigated how the emitted power of the V2V antenna(s) affects the exposure dose absorbed by the road user. In particular, we assessed the dose of exposure induced by V2V antennas operated at an emitted power level of either 33 dBm, which corresponds to the maximum emitted power allowed in the EU [7] or 44.8 dBm, which is the maximum limit in the USA [1].
4. The number of cars near the road user that are transmitting: we analyzed the impact of the presence of multiple transmitting cars in close proximity to the road user on the exposure levels.
5. The number of V2V antennas mounted on each transmitting car: we examined how the number of V2V antennas installed on each transmitting car influences the dose absorbed by the road user.

Regarding the variability due to the road user, our study investigated the absorbed dose in human models considering the following factors:

6. Body size: we analyzed how variations in body size, such as height and weight, impact the absorbed dose.
7. Age: we considered human models spanning different age groups, from children to adults, to assess how age affects the absorbed dose.
8. Gender: we examined the differences in absorbed dose between male and female human models.

To address the variability of the absorbed dose in realistic urban conditions, we developed and implemented a novel hybrid procedure that combines both deterministic and stochastic approaches. In the deterministic approach, we employed analytical models to simulate the propagation of the electromagnetic field in V2V communications within urban environments. This enabled us to obtain the exposure field and the resulting absorbed dose for various distances from the transmitting car(s). In the stochastic approach, we incorporated the typical variability observed in real urban layouts to account for the fluctuations and unpredictability of propagation conditions that are encountered in realistic urban scenarios.

The paper is organized as follows: in Section 2.1, we describe the metric we used to assess the exposure level in the human models investigated in our study; in Section 2.2, we outline the deterministic analytical approach we applied to calculate the exposure field at various distances from the transmitting car(s); in Section 2.3, we illustrate the stochastic approach we applied to account for the variability of the propagation conditions that are encountered in urban scenarios; in Section 2.4, we provide a description of the sources of variability that we addressed in relation to the absorbed dose; in Section 2.5, we illustrate the exposure scenarios we investigated in our study; in Section 2.6, we outline the descriptive statistics we used to analyze the absorbed dose calculated with the proposed hybrid approach; in Section 3 and Section 4, we present and discuss the results; and, finally, in Section 5, we draw the conclusions.

## 2. Methods

### 2.1. Calculation of the Absorbed Dose

The RF exposure in the road user was assessed by calculating the specific energy absorption rate (SAR) over the whole-body (SAR_wb_), which is the power of the RF electromagnetic field (RF-EMF) absorbed over the entire mass of the body. The evaluation of the SAR allowed us to assess if the dose of the RF-EMF absorbed by the human body was below the basic restrictions limits recommended by the International Commission on Non-Ionizing Radiation Protection (ICNIRP) [12] and the Institute of Electrical and Electronics Engineers (IEEE) [13] to protect against potential adverse health effects in the 100 kHz–300 GHz range. The exposure was assessed at the frequency of 5.9 GHz used in V2V vehicular connectivity [1,7]. 

Considering a road user of body mass index BMI_ru_ (in kg/m^2^), SAR_wb_ (W/kg) can be calculated based on the incident electric field E_inc_ (expressed as the root mean square value of the electric field measured in V/m) evaluated at the position of the road user. The formula for calculating SAR_wb_ is as follows [14]:

(1) $${\mathrm{SAR}}_{\mathrm{wb}}={\left(E_{\mathrm{inc}}/E_{\mathrm{ref}}\right)}^{2}\cdot \left({\mathrm{BMI}}_{\mathrm{ref}}/{\mathrm{BMI}}_{\mathrm{ru}}\right)\cdot {\mathrm{SAR}}_{\mathrm{ref}},$$

where SAR_ref_ (in W/kg) is the whole-body SAR induced by a reference incident field E_ref_ (in V/m) in a reference human body of body mass index BMI_ref_ (in kg/m^2^). The above formula assumes far-field conditions, where the distance between the road user and the transmitting source is large enough to be considered in the far-field region. At the frequency of 5.9 GHz, considering the typical dimensions of V2V antennas, the exposure of road users can be assumed to be in the far-field region. In fact, the typical distance between a road user and the nearest car’s V2V antenna is always greater than the far-field distance, which is calculated as 2D²/λ (where D is the most relevant dimension of the antenna, that for a V2V antenna is the length). For a typical V2V antenna with a length of 0.1 m and operating at 5.9 GHz (with a wavelength λ of 0.05 m), the far-field distance is 0.39 m.

The whole-body specific absorption rate (SAR_ref_) of the reference human body in Equation (1) is defined as follows [12]:

(2) $${\mathrm{SAR}}_{\mathrm{ref}}=\left(P_{\mathrm{wb},\mathrm{ref}}/M_{\mathrm{ref}}\right)=\left(1/M_{\mathrm{ref}}\right)\cdot \int_{\mathrm{wb}}^{}{\sigma (r)\;E}_{\mathrm{RMS}}^{2}(r)\;\mathrm{dV}$$

where P_wb,ref_ (in W) and M_ref_ (in kg) are the whole-body absorbed power and the mass of the reference human body, s (in S/m) is the electrical conductivity of the reference human body, E_RMS_ (in V/m) is the root mean square value of the electric field E induced in the reference human body, and V is the volume (in m^3^) occupied by the reference human body.

In our study, we utilized the SAR_ref_ values calculated by [14] through computational dosimetry in anatomical human models and determined in far-field conditions. The reference field E_ref_ used in [14] was equal to 2.45 V/m. These SAR_ref_ values provide a reference for assessing the absorbed dose in human models under far-field exposure conditions, allowing for comparisons and analysis across different scenarios and studies.

The SAR_ref_ values provided by [14] were calculated at a frequency of 5.8 GHz, which is slightly different from the nominal frequency of 5.9 GHz used in vehicular communication. It is to note that the dielectric properties of human tissues [15,16], which play a crucial role in determining the absorption of electromagnetic fields by the human body, are very similar at 5.8 GHz and 5.9 GHz. As a matter of fact, the conductivity at 5.8 GHz is, on average across all the tissues, 0.98 times than that at 5.9 GHz. Additionally, the relative permittivity at 5.8 GHz is, on average, the same as that at 5.9 GHz. As evidenced in [17], variations in dielectric properties up to a ratio of 2.0 do not substantially influence the whole-body SAR. Thus, due to the close proximity of these two frequencies, considering that 5.9 GHz is the nominal center frequency of the actual band used in vehicular communication (ranging from 5.855 to 5.925 GHz) and that the differences of the dielectric properties of the human tissues between the two frequencies are relatively small, the SAR _ref_ values from [14] can be considered reliable approximations of the SAR_ref_ values that would have been obtained at precisely 5.9 GHz.

### 2.2. The Deterministic Approach for the Calculation of the Incident Electric Field E_inc_ at the Road User Position

In the current Section, we describe the deterministic approach we applied to calculate the incident electric field E_inc_ to be used in Equation (1). We used the analytical model of V2V field propagation developed by [18]. This model allows the calculation of the received power and E_inc_ at any given distance from a V2V transmitting antenna. The model was validated in [18] by comparing the received power calculated using the model with the power measured at a V2V receiving antenna on a car while driving in real cities.

The approach in [18] accounts for the two principal propagation mechanisms of the exposure field in V2V communication, namely, the *large-scale signal variation* and the *small-scale signal variation*. For the *large-scale signal variation*, the model of [18] distinguishes three types of field propagation, namely, the propagation in Line-of-Sight (LOS) condition that occurs when the optical path between the transmitter (the car) and the receiver (the road user, in our study) is unobstructed, the non-LOS condition due to vehicles (NLOS_v_) when the optical path is obstructed by other vehicles, and the non-LOS condition due to buildings and foliage (NLOS_b_) when the path is obstructed by buildings or foliage. In [18], the propagation under LOS conditions was modelled with the two-ray ground reflection model [19], the attenuation due to vehicles along the LOS optical path in NLOS_v_ propagation conditions was modeled with the multiple knife-edge diffraction model [20], and the attenuation due to buildings under NLOS_b_ propagation conditions was modeled with the log-distance path loss model, as described in [21].

The analytical model of [18] also considers the *small-scale signal variation* due to multipath propagations, scattering, Doppler spread, and the variations due to the different type and shape of the obstructing objects (e.g., vehicles, buildings, and foliage). The *small-scale signal variation* was modeled by [18] as a zero-mean normally distributed signal, whose standard deviation σ depends on the type of the propagating condition (i.e., LOS, NLOS_v_, or NLOS_b_) and the number and density of cars and static objects in the area around the transmitter–receiver pair. In our study, we utilized the standard deviation σ estimated by [18], which was derived from measurements conducted in an urban environment, specifically Porto downtown. This standard deviation represents the variability observed in each propagation condition, taking into account real-world measurement data. The model equations are reported in detail in Appendix A of the paper (see Equations (A1)–(A4)).

We obtained the overall received power at any given position of the road user by adding the contribution of the *large-scale* and the *small-scale signal variation*. We calculated the overall power by varying the distance d_i_ (i = 1, …, 299) between the road user and the transmitting car within the range of 1.5–300 m, with a step size of 1 m.

### 2.3. The Stochastic Approach to Account for the Variability of the Propagation Conditions in Real Urban Settings

The field propagation condition (i.e., LOS, NLOS_v_, NLOS_b_) depends on the geometry of the area of interest around the road user, that is, on the position and outlines of buildings and roads and the position and number of the vehicles along the optical path between the transmitting car and the road user. If the geometry of the area of interest changes, e.g., by changing the position of the surrounding vehicles and the outlines of the buildings and roads, the propagation condition and the resulting E_inc_ at the position of the road user would change.

In the present work, we used a statistical approach to model the variability of the propagation condition in urban settings. We used the probability functions developed in [22], which provide the probabilities of the LOS, NLOS_v_, and NLOS_b_ propagation conditions as a function of the distance between the transmitting car and the receiver (i.e., the road user in our study). These probability functions were developed in [22] through fitting analytical functions to the path loss conditions observed in the downtown areas of Rome, London, Paris, Munich, New York, and Tokyo. By analyzing and measuring the path loss in these specific urban environments, the authors in [22] derived mathematical functions that accurately represent the observed variations in propagation conditions. The analytic expressions of such probability functions are reported in the Appendix A (see Equations (A5) and (A6)).

In our study, to account for the variability of the propagation conditions, we used the probability functions described above to generate 1000 instances of the propagation condition for each distance d_i_ (i = 1, …,299). In this way, we obtained 1000 × 299 random variations of the propagation condition that simulate the variability of the propagation condition that would be found in real urban layouts at different distances from the transmitting car. By applying the analytical model described in Section 2.2, we determined the incident electric field E_inc_ for each of the 1000 × 299 random propagation conditions. Finally, we obtained the corresponding SAR_wb_ using Equation (1), where we utilized the calculated E_inc_. We assumed that the road user was standing alongside the roads; we calculated E_inc_ at the height of the head of each human model. 

### 2.4. Sources of Variability of SARwb

In the following paragraphs, we explain how we took into account the sources of variability of SAR_wb_. The variability of SAR_wb_ is primarily influenced by three main factors, i.e., the anatomical characteristics of the road user, the different urban layouts, and the operational conditions of V2V communication. The anatomical characteristics of the road user directly impact SAR_wb_. These characteristics include factors such as gender, body size, and age (as evidenced for example in [23,24,25,26]). On the other hand, the urban layout and the operational conditions of the V2V antennas have a more global impact on E_inc_, which in turns affects SAR_wb_. For the urban layouts, factors such as road and building geometries and the position, size, and number of cars in the scene can introduce variability in the propagation conditions and consequently influence E_inc_. Similarly, the operational conditions of V2V communication, such as power levels, gain and height of the emitting antennas, can affect the overall strength and distribution of E_inc_ in the environment.

More specifically, as described in the Appendix A (see Equations (A1)–(A3)), E_inc_ depends on: the distance between the road user and the transmitting car; the operating conditions of the transmitting antenna(s); the height of the receiver; the height of the surrounding cars; and, finally, the propagation condition along the optical path between the transmitting car(s) and the road user. For the variability of the propagation condition, we preferred for sake of clarity to address this important aspect in a separate Section (see previous Section 2.3).

#### 2.4.1. Variability Due to the Characteristics of the Road User

As described in Section 2.1, we calculated the exposure induced by V2V communication by using the SAR_ref_ values calculated in [14] in reference human models. These latter human models belong to the Virtual population ViP2.0 (https://itis.swiss/virtual-population/virtual-population/overview/ accessed on 29 July 2023) of anatomical models developed from a collection of high-resolution Magnetic Resonance Imaging (MRI) data [27]. We considered in our study five human models from [14], whose characteristics and the corresponding SAR_ref_ values are displayed in Table 1.

We calculated SAR_wb_ induced by V2V communication in the five models described above by setting BMI_ru_ = BMI_ref_ in Equation (1).

#### 2.4.2. Variability Due to the Distance from the Transmitting Car

To account for the variability due to the different positions of the road user, we calculated E_inc_ (and, consequently, SAR_wb_) for distances d_i_ (i = 1, …,299) between the road user and the transmitting car ranging from 1.5 to 300 m, with a step of 1 m.

#### 2.4.3. Variability Due to the Operating Conditions of the V2V Antenna(s)

For the transmitting antenna(s), we considered two levels of the emitted power: 33 dBm (1.99 W), which corresponds to the maximum power allowed in the EU [7], and 44.8 dBm (30.2 W), which is the maximum limit in the USA [1]. E_inc_ and the resulting SAR_wb_ were calculated based on the assumption that the V2V antenna(s) were mounted on the roof of passengers cars. As a result, the placement of the antennas was considered at a height of 1.6 m from the ground. Finally, the antennas were assumed to have a gain equal to 0 dB.

#### 2.4.4. Variability Due to the Height of the Receiver

For the variability due to the height of the receiver, i.e., the road user, we calculated E_inc_ at the height of the heads of the five human models, that is, 0.92 m for ‘Nina’, 1.07 m for ‘Thelonious’, 1.48 m for ‘Billie’, 1.60 m for ‘Ella’, and 1.70 m for ‘Duke’.

#### 2.4.5. Variability Due to the Height of the Surrounding Cars

As described above, the vehicles surrounding the transmitting car may act as obstacles for the propagation of the field generated by the transmitting car. In such a case, the propagation condition is the NLOS_v_ type. The height of surrounding vehicles is a significant variable in determining the level of attenuation in the field emitted by the transmitter under such NLOS_v_ propagation conditions (see Equation (A2) in the Appendix A). To account for the variability in the height of surrounding cars, we incorporated this aspect as a random variable in our analysis. Specifically, we modeled the height of the surrounding cars as a normal distribution with a mean of 1.5 m and a standard deviation of 0.15 m, which aligns with the typical height range observed in passenger cars [18].

### 2.5. The Analyzed Exposure Scenarios

For each human model, we calculated the distribution of SAR_wb_ in four distinct exposure scenarios, whose main characteristics are resumed in Table 2.

In scenario A and B, we analyzed the exposure generated by a single transmitting car. In line with the typical V2V antennas’ montage guidelines, we assumed that each transmitting car could be equipped with up to four V2V antennas. Each antenna was operated at an emitted power level of 33 dBm in scenario A and at 44.8 dBm in scenario B. In both scenarios, we calculated the distribution of SAR_wb_ values for the 1000 × 299 random propagation conditions for distances d_i_ in the 1.5–300 m range (see Section 2.2 and Section 2.3).

In scenario C and D, we assessed SAR_wb_ generated with more than one transmitting car equipped with V2V antennas operated at an emitted power level of 33 dBm in scenario C and 44.8 dBm in scenario D. We considered in the area of interest around the road user the presence of up to N_TX_ transmitting cars, each equipped with one to four V2V antennas. To calculate SAR_wb_, we first generated an array D_TX_ of (1000 × N_TX_) random distances between the road user and each transmitting car. Basically, this procedure allowed us to simulate the presence of N_TX_ transmitting cars in the area of interest at 1000 random distances from the road user. Then, for each distance in the array D_TX_, we determined the propagation condition following the procedure described in Section 2.3 and the corresponding E_inc_ with the procedure described in Section 2.2. To calculate the resulting SAR_wb_ induced by the N_TX_ transmitting cars, we determined the SAR_wb_ generated by each car individually. Afterwards, we summed together the SAR_wb_ values for each car to obtain the overall SAR_wb_ induced by the N_TX_ transmitting cars.

### 2.6. Calculation of the SAR_wb_ Descriptive Statistics

For each scenario, we calculated the median and the 1st, 25th, 75th, and 99th percentiles of the distributions of SAR_wb_ obtained from varying the distance between the transmitting car(s) and the road user.

## 3. Results

**Figure 2:**
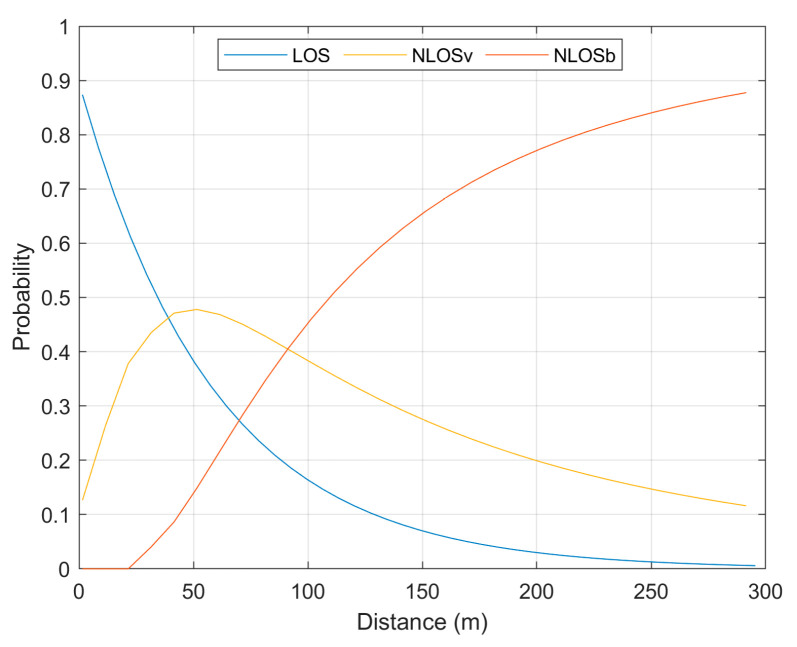
Probabilities of LOS, NLOS_v_, and NLOS_b_ propagation conditions along the optical path between a transmitting car and a road user in urban settings as a function of the distance d_i_ from the transmitting car.

Figure 2 shows the probabilities for LOS, NLOS_v_, and NLOS_b_ propagation conditions in urban settings, as calculated with the probability functions of [20] (see Section 2.3 and also Equations (A5) and (A6) in the Appendix A).

At any given distance d_i_ from the transmitting car, the sum of the three probabilities for the LOS, NLOS_v_, and NLOS_b_ propagation conditions is one. The probability for the LOS propagation condition progressively decreased with the distance, meaning that at shorter distances it is highly probable that the optical path between the car and the road user would be unobstructed, whereas at longer distances, the LOS propagation condition becomes less likely due to a higher probability of obstructions such as buildings and surrounding cars blocking the optical path. The probability for the NLOS_v_ propagation condition, that is, the probability that the optical path between the transmitting car and the road user would be obstructed by another vehicle, was at its maximum (equal to a probability of nearly 0.5) at about 50 m from the transmitting car. The NLOS_b_ was the most probable propagation condition at distances longer than 100 m. On the contrary, at distances shorter than 100 m, the LOS and NLOS_v_ were the most probable propagation conditions.

**Figure 3:**
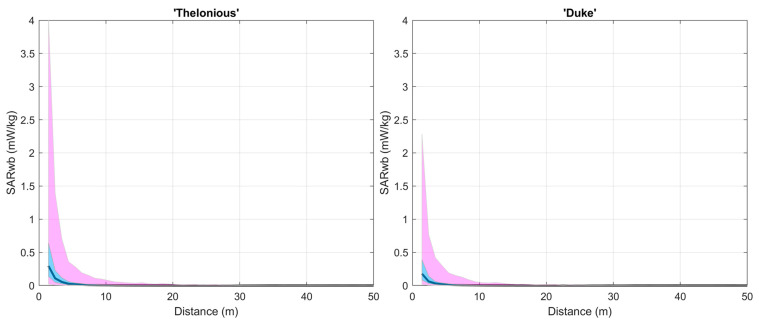
SAR_wb_ for one child model (‘Thelonious’) and one adult model (‘Duke’) as a function of the distance d_i_ from the transmitting car. The car was equipped with one antenna operated at an emitted power level of 33 dBm. SAR_wb_ was calculated in far-field conditions for an incident electric field E_inc_ evaluated at the level of the head of each model, that is, at 1.07 m for ‘Thelonious’ and 1.70 m for ‘Duke’. For sake of clarity, the figure shows the SAR_wb_ values only for distances up to 50 m because for greater distances SAR_wb_ was negligible. The bold line is the median; the pink-shaded area is the 1st–99th percentile area; the cyan-shaded area is the 25th–75th percentile area.

As an example, Figure 3 shows the distribution of SAR_wb_ as a function of the distance d_i_ for the child model ‘Thelonious’ and the adult model ‘Duke’. The SAR in Figure 2 corresponds to the exposure induced by only one transmitting car equipped with a single antenna operated at an emitted power level of 33 dBm.

As expected, it is observed in Figure 3 that SAR_wb_ decreased with the distance between the road user and the transmitting car. The greatest differences of SAR_wb_ between the adult and the child models were observed at the shortest distances from the car, with SAR_wb_ being higher in the child than in the adult model.

### 3.1. Exposure Dose in Scenario A—One Transmitting Car with Antennas Operated at 33 dBm

Table 3 displays for all the human models the descriptive statistics of the SAR_wb_ distributions of the exposures generated by a single car equipped with either one (scenario A1) or four emitting V2V antennas (scenario A2). In both scenarios, each antenna was operated at an emitted power level of 33 dBm. To calculate the SAR_wb_ values for scenario A2, the SAR_wb_ values obtained in scenario A1 for the exposure generated by a single antenna were multiplied by a factor of four to account for the presence of the four antennas. It is important to note that the SAR_wb_ calculations were performed under far-field conditions. In this context, we made the assumption as if the four antennas were placed at the center of the roof of the transmitting car. This assumption implies that variations in the positions of the antennas on the car roof do not significantly affect the exposure levels. 

To facilitate the comparison of exposure due to the change in the number of emitting antennas, the SAR_wb_ distributions in Table 3 were evaluated for both scenario A1 (one emitting antenna) and A2 (four emitting antennas) for distances d_i_ within the same range. This range, denoted as d_lim_, represents the distance beyond which the SAR_wb_ observed in scenario A1, i.e., for exposure generated by a single transmitting car, with one antenna operated at an emitted power level of 33 dBm, becomes lower than 1% of the basic restriction limit for the whole-body SAR [12,13].

As observed in Table 3, the median value of SAR_wb_ across the various human models and for distances within d_lim_ was found to be quite low, ranging from 0.02 mW/kg with one emitting antenna to 0.32 mW/kg with four emitting antennas. 

To get an insight into the potential highest exposure levels in the studied scenarios, we analyzed the 99th percentile value of SAR_wb_. The 99th percentile across the various human models ranged from 0.35 mW/kg (with one emitting antenna) to 8.59 mW/kg (with four emitting antennas). It was observed that the 99th percentile of SAR_wb_ changed with the body mass and height of the human model. Specifically, it tended to be inversely related to the body mass of the model, being lower for the models with higher body mass. This trend was observed in all human models apart from the shortest one, i.e., ‘Nina’. As a matter of fact, SAR_wb_ in ‘Nina’ was smaller than in all the other models, although this model had a quite low body mass of 13.9 kg. This might be because the height of the model ‘Nina’ (0.92 m) was significantly below the height of the antenna (1.6 m); as such, it is highly probable that this particular model experienced only marginal exposure to the field emitted by the antenna(s), resulting in a lower SAR_wb_ compared to the other human models. The other models, instead, were at a height ranging from 1.07 m to 1.7 m, which was more similar to the height of the antenna, and we assumed it to be equal to 1.6 m; as such, these latter models could be exposed to the field radiated by the antenna(s) more than ‘Nina’.

We found that the absorbed dose in human models was not impacted by gender or age. As a matter of fact, when comparing human models of the same size, regardless of their gender or age, the absorbed dose remained the same.

As a final remark, for all human models, SAR_wb_ was in any case below the basic restriction limit of 80 mW/kg for the whole-body SAR [12,13], even considering the SAR values corresponding to the 99th percentile of the distribution.

### 3.2. Exposure Dose in Scenario B—One Transmitting Car with Antennas Operated at 44.8 dBm

Table 4 displays the descriptive statistics of the SAR_wb_ distribution obtained with a single transmitting car equipped with one (scenario B1) vs. four V2V emitting antennas (scenario B2). In contrast to Table 3, the values of SAR_wb_ in Table 4 were calculated by assuming that each antenna was operated at an emitted power level of 44.8 dBm instead of 33 dBm. The SAR_wb_ was obtained by scaling the distributions obtained in scenario A1 for one antenna operated at 33 dBm emitted power, by the emitted power level of 44.8 dBm and the number of emitting antennas. Table 4 reports for both scenarios B1 and B2 the distribution of SAR_wb_ for distances d_i_ within the same range d_lim_, which represents the distance beyond which the SAR_wb_ observed in scenario B1, i.e., for exposure generated by a single transmitting car, with one antenna operated at an emitted power level of 44.8 dBm, becomes lower than 1% of the basic restriction limit for the whole-body SAR [12,13].

As observed with antennas operated at 33 dBm (Table 3), also at an emitted power level of 44.8 dBm per antenna, the median value of SAR_wb_ within the distance d_lim_ remained consistently low, ranging from 0.02 mW/kg with one emitting antenna to 0.25 mW/kg with four emitting antennas. The 99th percentile of SAR_wb_ ranged from 1.97 mW/kg with one emitting antenna to 52 mW/kg with four emitting antennas, as observed in Table 4. Even when considering four emitting antennas, the 99th percentile of SAR_wb_ induced by a single transmitting car, with each antenna operated at an emitted power level of 44.8 dBm, did not exceed the established basic restriction limits of 80 mW/kg for the whole-body SAR [12,13]. 

### 3.3. The Impact of the Emitted Power and Number of Emitting Antennas on the Distance d_lim_

In the current Section, we delve deeper into the analysis of d_lim_, which represents the distance at which SAR_wb_ becomes lower than 1% of the basic restriction limit for the whole-body SAR [12,13]. Specifically, we investigated how d_lim_ changed across the different human models by varying both the emitted power levels and the number of emitting antennas. In Table 5, the values of d_lim_ are reported for exposures generated by a single car as a function of the emitted power level (33 dBm vs. 44.8 dBm) and the number of emitting antennas (one vs. four). Notably, in Table 5, the values of d_lim_ presented for exposure induced by one emitting antenna are the same as those already shown in Table 3 for 33 dBm emitted power and in Table 4 for 44.8 dBm emitted power.

As expected, it is possible to observe from Table 5 that for each human model the distance d_lim_ increased with the emitted power and the number of emitting antennas. This implies that as the power emitted by the antennas and the number of emitting antennas increase, SAR_wb_ remains significant for longer distances. At an emitted power level of 33 dBm per antenna, SAR_wb_ was significant for distances up to 8.5 m for exposures generated by one antenna and 13.5 m for exposures generated by four antennas. At an emitted power level of 44.8 dBm per antenna, SAR_wb_ was significant for distances up to 40.5 m for exposures generated by one antenna and 77.5 m for exposures generated by four antennas.

### 3.4. Exposure Dose in Scenario C—Multiple Transmitting Cars with Antennas Operated at 33 dBm

As described in Section 2.5, in a second step of our analysis, we focused on the impact of multiple transmitting cars in close proximity to the road user. In the current Section, we specifically describe the exposure induced by multiple transmitting cars with either one (scenario C1) or four antennas (scenario C2), and each operated at an emitted power level of 33 dBm.

In contrast to scenarios A and B, in this second step of our analysis, we decided to evaluate the exposure within the same distance limit for all the human models and for both scenario C1 and C2. Specifically, to calculate the exposure, we assumed that in scenario C1 and C2 each transmitting car was randomly positioned within the highest distance limit observed in Table 5 for exposure generated by one transmitting car equipped with antennas operated at 33 dBm emitted power level. This ensures a comprehensive assessment of the exposure within the chosen distance range for both scenario C1 and C2 and all the human models. At an emitted power level of 33 dBm per antenna, this ‘highest’ distance limit was determined to be equal to 13.5 m, as observed in Table 5 for exposures generated by four antennas. 

To determine the number of transmitting cars to be positioned within such a distance limit of 13.5 m, we considered two different vehicle density conditions, that is, 30 vehicles/mile per lane, which is the density corresponding to a regular flow of the vehicles, and 67 vehicles/mile per lane, which corresponds to unstable flow conditions characterized by traffic jams and stop-and-go driving conditions. Thus, assuming that the area around the road user consists of roads with two lanes of an overall length of 13.5 m, the maximum number N_TX_ of transmitting cars in such an area is equal to two cars at both vehicle densities.

Table 6 presents the SAR_wb_ values induced by N_TX_ = 2 transmitting cars placed at a variable distance within 13.5 m from the road user and equipped with antennas each operated at an emitted power level of 33 dBm.

Please note that Table 6 displays the descriptive statistics of the SAR_wb_ values calculated by varying the position of each of the transmitting cars within 13.5 m, for all human models and for both scenario C1 and C2. On the other hand, the SAR_wb_ distributions in Table 3 were calculated by varying the position of the (single) transmitting car within a distance limit, d_lim_, specific for each human model. As shown in Table 3, these latter distance limits ranged from 1.5 m to 8.5 m and were lower than the fixed distance limit of 13.5 m that we utilized to calculate the SAR_wb_ displayed in Table 6.

As observed in Table 6, the median value of SAR_wb_ generated by two transmitting cars placed at variable distances within 13.5 m from the road user remained consistently small across the human models and within 0.16 mW/kg. In contrast to the median, the 75th and 99th percentiles of SAR_wb_ changed across the diverse human models. As a general trend, the 75th and 99th percentiles showed a decrease with increasing body mass (with the exception of ‘Nina’). The highest value of the 99th percentile, equal to 7.08 mW/kg, was observed in the child model ‘Thelonious’ in scenario C2, which involved two transmitting cars with four emitting antennas. The lowest dose of exposure was observed in ‘Nina’ for which SAR_wb_ remained consistently below 0.7 mW/kg.

Among the children, the 99th percentile of SAR_wb_ showed the highest variability, especially in scenario C2. In this scenario, the 99th percentile ranged from 0.61 mW/kg to 7.08 mW/kg among the children. In contrast, the 99th percentile values were nearly identical across the adult models, with ‘Duke’ exhibiting 4.74 mW/kg and ‘Ella’ displaying 4.79 mW/kg. Across the different human models and different scenarios, the highest value of the 99th percentile of SAR_wb_, equal to 7.08 mW/kg, remained below the established basic restriction limits of 80 mW/kg for the whole-body SAR [12,13].

### 3.5. Exposure Dose in Scenario D—Multiple Transmitting Cars Car with Antennas Operated at 44.8 dBm

Table 7 reports the descriptive statistics of the exposure generated multiple transmitting cars with antennas operated at an emitted power level of 44.8 dBm. The exposure was calculated by assuming that each transmitting car was randomly placed at variable distance within the range of 77.5 m from the road user, which was the highest distance limit at which the SAR_wb_ induced by only one transmitting car with antennas operated at 44.8 dBm was significant, as shown in Table 5. As in scenario C, the number N_TX_ of transmitting cars within the range of 77.5 m was determined for two traffic densities, namely, at 30 and 67 vehicles/mile per lane. Thus, assuming that the area of interest was made of roads with two lanes and an overall length of 77.5 m, N_TX_ would be equal to three cars at the density of 30 vehicles/mile per lane and six cars at 67 vehicles/mile per lane. 

As observed in Table 7, the SAR_wb_ generated at an emitted power level of 44.8 dBm was always greater than that at 33 dBm (Table 6). As observed at 33 dBm, also at 44.8 dBm emitted power, the median SAR_wb_ remained consistently low, being at a maximum value equal to 0.70 mW/kg. The 99th percentile of SAR_wb_ significantly increased with the number of transmitting cars and antennas and was generally greater for the human models with lower body mass, namely, the 99th percentile of the SAR_wb_ generated across the different human models and the different scenarios ranged from 0.98 mW/kg up to 72.63 mW/kg. The variation of the 99th percentile was more pronounced across the children models (for which SAR_wb_ ranged from 0.98 mW/kg to 72.63 mW/kg) than in the two adult models (for which it ranged from 7.18 mW/kg to 48.82 mW/kg). In the current scenario D, the 99th percentile of SAR_wb_ remained below the established basic restriction limits of 80 mW/kg for the whole-body SAR [12,13].

## 4. Discussion

In the current study, we assessed RF-EMF exposure in road users by considering the variability of V2V exposure in typical urban conditions. Our study captured the variations in exposure caused by three key factors: i. different urban layouts, including the effects of the different position of the road user near the transmitting car(s) and the different position and size of the objects in the scene (i.e., buildings and surrounding cars), ii. different operational conditions of V2V communication, including the effects of the power emitted by the V2V antennas, the number of transmitting cars near the road user, and the number of antennas mounted on each car, and iii. different anatomical characteristics of road users by considering human models of both genders and different ages, ranging from children to adults.

We assessed the exposure by calculating the dose absorbed by the whole-body (SAR_wb_) in human models. To account for the variability of SAR_wb_ in realistic urban conditions, we developed a hybrid procedure that combines a deterministic approach with a stochastic approach. Specifically, we used a deterministic approach [18] to analytically model the propagation of the field in V2V communications in urban conditions and to obtain the corresponding SAR_wb_ at any given distance from the transmitting car and a stochastic approach [22] to simulate the typical variability of the propagation conditions observed in real urban layouts due to the presence of obstacles (i.e., buildings and other vehicles) of varying dimensions and at varying positions along the optical path between the transmitting car and the road user. As a result of the application of the proposed hybrid procedure, we were able to calculate the distributions of the SAR_wb_ values that accounted for the variability of the propagation conditions and the variability of the urban layouts. By using this hybrid approach, we were able to assess the level of exposure in different scenarios, rather than being limited to a single urban layout with specific geometry. On the contrary, the deterministic approach would have only provided results for the specific urban layout being considered.

In the current study, we performed a comparison of the SAR_wb_ obtained with the currently available basic restrictions limits for whole-body exposure set by the ICNIRP [12] and IEEE [13]. These limits were established to assess the whole-body exposure level averaged over a duration of 30 min, with the primary objective of safeguarding against a potential rise in body core temperature of 1 °C. The decision to use a 30-min averaging time, chosen by the ICNIRP and IEEE, was justified by the need to account for the time required to reach a steady-state temperature within the body.

In our analysis of SAR_wb_ in urban settings, we considered the exposure generated by both a single transmitting car and multiple transmitting cars. For the exposure generated by a single transmitting car, we evaluated the distribution of the SAR_wb_ values within a specific distance limit for each human model. Specifically, we defined it as the distance beyond which the exposure induced in each human model by one transmitting car with one emitting antenna become negligible (i.e., become lower than 1% of the basic restriction limit for whole-body exposure [12,13]). Such distance limit ranged across the human models from 1.5 to 8.5 m for exposures generated at 33 dBm emitted power and from 8.5 to 40.5 m for exposures generated at 44.8 dBm emitted power. Overall, the median value of SAR_wb_ induced by a single transmitting car within these latter distance limits remained consistently low, being at maximum 0.32 mW/kg across the human models and for both levels of emitted power.

In addition to the median value, we focused our attention on the 99th percentile value of SAR_wb_ as it provides valuable insight into the potential highest exposure levels observed across the different scenarios analyzed. When only one car is transmitting, the 99th percentile of SAR_wb_ was at a maximum value equal to 8.59 mW/kg for exposures generated at 33 dBm emitted power and 52 mW/kg for exposures generated at 44.8 dBm emitted power.

To evaluate the impact of multiple transmitting cars near the road user, we performed SAR_wb_ calculations considering two distinct traffic conditions: normal traffic density and high traffic density. The evaluation of the exposure from multiple transmitting cars was conducted within a longer distance limit compared to the distance limit used to evaluate the exposure generated by a single transmitting car. Specifically, for cars equipped with antennas operated at 33 dBm emitted power, the evaluation was performed within a distance limit of 13.5 m; for antennas operated at 44.8 dBm emitted power, the evaluation was extended to a distance limit of 77.5 m. At normal traffic density and with an emitted power level of 33 dBm, both the median and the 99th percentile of exposure from multiple cars placed at varying distances within 13.5 m were found to be of the same order of magnitude as those observed at a closer distance within 8.5 m from a single emitting car. Similarly, at the higher emitted power level of 44.8 dBm, the median and the 99th percentile of exposure from multiple transmitting cars placed at varying distances within 77.5 m were similar to those observed at a closer distance within 40.5 m from a single emitting car. On the contrary, at high traffic density and at an emitted power level of 44.8 dBm, the 99th percentile of exposure when multiple cars are transmitting can be up to 1.4 times higher than the exposure observed within 40.5 m when only one car is transmitting.

Overall, based on the analysis conducted in the current study, considering all the scenarios and conditions (i.e., including the scenarios with multiple transmitting cars), we found that the 99th percentile of SAR_wb_ could reach a maximum of 8.59 mW/kg at 33 dBm emitted power, whereas at the higher level of emitted power of 44.8 dBm, the 99th percentile of SAR_wb_ could reach a maximum of 72.63 mW/kg.

We observed significant variation in the 99th percentile of SAR_wb_ among different human models. Specifically, for all human models with a height greater than 1 m, we noticed a decrease in SAR_wb_ as the body mass increased. In particular, we observed that the adults, being heavier than the children, were exposed to a lesser dose of RF-EMF than the children. The smallest and youngest model (‘Nina’) whose anatomical characteristics resembled those of a three years old child of 0.92 m height was an exception. As a matter of fact, although this was the model with the lowest body mass, the 99th percentile of its SAR_wb_ was lower than that observed in all the other models. It is to note that the height of this young child model was below the height of the V2V antennas (equal to 1.6 m); as such, it is highly probable that this particular human model experienced only marginal exposure to the field emitted by the antenna(s), resulting in a lower SAR_wb_ compared to the other human models. This effect of the body mass on the SAR, with SAR_wb_ being lower in subjects with higher body mass, was in line with both the theory (as seen in Equation (2), the SAR is inversely proportional to the body mass) and with what was observed in previous numerical dosimetry studies, e.g., in [14].

Finally, in the context of our study and the specific parameters we considered, our findings indicated that gender and age did not have a significant influence on the absorbed dose. As a matter of fact, when comparing human models of the same size, regardless of their gender or age, the absorbed dose remained the same.

To our knowledge, this is the first study investigating the variability of the exposure to RF arising from V2V communication in realistic urban settings. A recent survey conducted in [28] indicates that there is a limited amount of research (see, e.g., [8,9,10,11]) available on the assessment of RF human exposure in vehicular communication scenarios. The authors in [11] investigated the dose of exposure for a passenger (an adult human model) within a car equipped with four V2V antennas operated at 5.9 GHz. The exposure was evaluated through numerical dosimetry by using the same human model ‘Duke’ as in the current work. The study in [11] evidenced that when all the four antennas were operated at the same time at 44.8 dBm emitted power each, the whole-body SAR of the car passenger was equal to 8.33 mW/kg. This latter value is higher than the 0.15 mW/kg median value of SAR_wb_ that we observed in the same model ‘Duke’ in outdoor urban scenarios with one transmitting car and four antennas operated at 44.8 dBm emitted power. On the contrary, the 99th percentile of SAR_wb_ of ‘Duke’ that we observed in outdoor urban scenarios, equal to 29.67 mW/kg, was higher than the SAR calculated in [11] for the same model inside the car. As a general remark, also when multiple cars are transmitting, the median value of SAR_wb_ in outdoor urban scenarios was always below the SAR inside the car, whereas the 99th percentile value of the SAR outdoor was higher than that inside the car.

Furthermore, the authors in [8,9] investigated the exposure from 5.9 GHz V2V communication in human models placed outdoor, in close proximity of the transmitting car. The method applied in these two latter studies was again a deterministic approach, based on numerical dosimetry, as in [11]. Two of the models analyzed in [8,9], i.e., the child ‘Thelonious’ and the adult ‘Ella’, were the same as in our current study. In [8,9], the human models were positioned at the closest possible distance from the body of the car, ensuring that no physical contact was made with the car’s body. This setup provided an estimation of the exposure that would be observed outdoors but at the closest proximity to the car. In contrast, in our study, the human models were assumed to be positioned at greater distances from the transmitting car(s), ranging from 1.5 to 300 m. Therefore, the results obtained in [8,9] estimated the whole-body SAR at the closest distance from the car, whereas the results from the current study provided an estimate of the SAR in a larger area around the transmitting car(s). It is noteworthy that even when considering exposures due to multiple emitting antennas, the median SAR_wb_ observed in urban scenarios in the current study was lower than the whole-body SAR evaluated at the closest distance from the car, as reported in [8,9] for both models (child and adult). However, the whole-body SAR at the closest distance from the car was lower than the 99th percentile of SAR_wb_ observed by us in the larger area around the car.

Based on our findings, on average, outdoor exposure arising from V2V communication in urban scenarios tended to be lower than exposure inside or at the closest distance from the transmitting car. However, it is important to note that due to the variability of the urban layout and other factors, there can be instances where outdoor exposure, specifically at the 99th percentile (extreme cases), may be significantly higher than exposure inside or at the closest distance from the car. These extreme instances highlight the potential for localized areas or specific conditions where outdoor exposure can exceed in-vehicle or close proximity exposure levels. It underscores the importance of considering the variability and specific circumstances when evaluating exposure levels in urban environments.

Indeed, further studying the variation of the effects of radiation as a function of the frequency band at which vehicular communication technology operates could provide valuable insights. Such studies can contribute to the development of communication systems that minimize RF exposure while maintaining effective communication performance. 

To the best of the authors’ knowledge, there is only one study [10] that has investigated the exposure at frequencies different from 5.9 GHz. The study in reference [10] investigated the exposure generated by the novel 5G-V2X technology operating in the FR1 band at 3.5 GHz. Through a deterministic dosimetry approach, the study assessed the exposure caused by a transmitting car equipped with two Uniformly Linear Array antennas, with eight transmitting elements each, operating at a power level of 30 dBm (1 W). The results showed that the exposure in an adult female human model remained below the safety limits set by the ICNIRP [12] and IEEE [13] guidelines. The SAR_wb_ values induced by the two array antennas ranged from 0.001 mW/kg to 0.074 mW/kg, depending on the position of the human model around the transmitting car. Scaling the results to the emitted power levels considered in the current study, the SAR_wb_ values calculated in [10] would equate to 0.002–0.15 mW/kg at a power level of 33 dBm and 0.03–2.23 mW/kg at a power level of 44.8 dBm. In comparison, based on the results obtained in the current study, using one transmitting car as shown in Table 3 and Table 4, the SAR_wb_ generated in the female model ‘Ella’ by two transmitting antennas operating at 5.9 GHz would be within 1.96 mW/kg at an emitted power level of 33 dBm and 18.60 mW/kg at an emitted power level of 44.8 dBm. These findings suggest that the exposure levels calculated in the current study for the specific V2V 5.9 GHz communication scenario align with the results from the previous study [10] at different frequency band (3.5 GHz) and different technology (5G-V2X operated through array antennas). The exposures remain within the safety limits established by international guidelines. Further research investigating other frequency bands and their corresponding exposure levels could offer a more comprehensive understanding of the safety implications of vehicular communication technologies.

As a last remark, it is noteworthy that there is the need for additional research to gain a comprehensive understanding of the variability of exposure in vehicular communication. Specifically, it would be interesting to expand the investigation beyond the V2V communication scenario discussed in the current study by also including the other types of vehicular communications scenarios, such as vehicle-to-infrastructure communication, vehicle-to-network communication, and vehicle-to-pedestrian communication. By studying these different aspects, a more complete understanding of the exposure in vehicular communication can be achieved.

## 5. Conclusions

This study investigated for the first time the variability of the RF-EMF dose absorbed at the whole-body of road users of different age and both genders in realistic urban conditions in V2V communications.

We found that the absorbed dose remained below the safety limits set by the ICNIRP [12] and IEEE [13], which specify a maximum limit of 80 mW/kg for whole-body human exposure to EMF. The absorbed dose remained below these limits even in vehicular communication scenarios with multiple transmitting cars and multiple antennas.

For the variability of the exposure, the median value of the distribution of the whole-body SAR across the different positions and distances from the transmitting car and the different variations of the urban layout was always very low, being as high as 0.70 mW/kg, even in scenarios with multiple transmitting cars and multiple emitting antennas per car. Interestingly, we observed that the 99th percentile of SAR_wb_ could be much more higher than the median value, being as high as nearly 73 mW/kg. This means that, from a statistically point of view, there could be some circumstances in which the dose absorbed by the road user could be high, but in any case below the basic restriction limits.

## Figures and Tables

**Table 1:** The characteristics and SAR_ref_ values of the five human models analyzed in our study. The SAR_ref_ values shown in the current table are the whole-body SAR calculated by [14] in far-field propagation conditions at 5.8 GHz and obtained for an incident electric field of 2.45 V/m.

Variable	Model Name				
	Nina	Thelonious	Billie	Ella	Duke
Sex	female	male	female	female	male
Age (yrs)	3	6	11	26	34
Body mass (kg)	13.9	19.5	34.6	58.7	72.4
Height (m)	0.92	1.07	1.48	1.60	1.70
BMI (kg/m^2^)	16.4	14.8	15.5	22.7	23.1
SAR_ref_ (W/kg)	6.0 × 10^−6^	6.4 × 10^−5^	5.1 × 10^−5^	4.0 × 10^−5^	3.6 × 10^−5^

**Table 2:** The main characteristics of the four exposure scenarios analyzed in our study.

Scenario	Number of Transmitting Cars	Number of V2V Antennas Per Car	Power Emitted by Each Antenna (dBm)
A	1	1 to 4	33
B	1	1 to 4	44.8
C	more than 1	1 to 4	33
D	more than 1	1 to 4	44.8

**Table 3:** SAR_wb_ (in mW/kg) of the five human models for distances d_i_ within d_lim_ from a car equipped with either one (scenario A1) or four V2V emitting antennas (scenario A2), with each antenna operated at an emitted power level of 33 dBm (1.99 W). The distance d_lim_ reported in the current Table represents the distance beyond which the SAR_wb_ generated by a single transmitting car, with a single antenna operated at an emitted power level of 33 dBm, became lower than 1% of the basic restriction limit of the whole-body SAR [12,13].

Human Model	d_lim_	SAR_wb_ (mW/kg)	
		Scenario A1: One Car, One Antenna	Scenario A2: One Car, Four Antennas
Nina	1.5 m	0.02 ^(*)^ (0 ^†^–0.35) ^(**)^ (0.01–0.05) ^(§)^	0.10 (0.01–1.42) (0.05–0.21)
Thelonious	4.5 m	0.08 (0 ^†^–2.15) (0.03–0.23)	0.32 (0 ^†^–8.59) (0.12–0.91)
Billie	6.5 m	0.04 (0 ^†^–1.57) (0.01–0.12)	0.15 (0 ^†^–6.27) (0.05–0.49)
Ella	8.5 m	0.02 (0 ^†^–0.98) (0.01–0.06)	0.07 (0 ^†^–3.91) (0.02–0.25)
Duke	5.5 m	0.04 (0 ^†^–1.24) (0.01–0.10)	0.15 (0 ^†^–4.95) (0.05–0.41)

^(*)^ median; ^(**)^ 1st–99th percentile; ^(§)^ 25th–75th percentile; ^†^ <10^−2^ mW/kg.

**Table 4:** SAR_wb_ (in mW/kg) of the five human models for distances d_i_ within d_lim_ from a car equipped with either one (scenario B1) or four V2V antennas (scenario B2), with each antenna operated at an emitted power level of 44.8 dBm (30.2 W). The distance d_lim_ reported in the current Table represents the distance beyond which the SAR_wb_ generated by a single transmitting car, with a single antenna operated at an emitted power level of 44.8 dBm, became lower than 1% of the basic restriction limit of the whole-body SAR [12,13]. Other details as in previous Table 3.

Human Model	d_lim_	SAR_wb_ (mW/kg)	
		Scenario B1: One Car, One Antenna	Scenario B2: One Car, Four Antennas
Nina	8.5 m	0.04 (0 ^†^–1.97) (0.01–0.13)	0.15 (0 ^†^–7.89) (0.04–0.50)
Thelonious	21.5 m	0.06 (0 ^†^–13.00) (0.01–0.29)	0.25 (0 ^†^–52.00) (0.06–1.17)
Billie	40.5 m	0.02 (0 ^†^–7.25) (0 ^†^–0.07)	0.06 (0 ^†^–29.00) (0.02–0.30)
Ella	19.5 m	0.06 (0 ^†^–9.30) (0.02–0.24)	0.24 (0 ^†^–37.21) (0.07–0.96)
Duke	24.5 m	0.04 (0 ^†^–7.42) (0.01–0.15)	0.15 (0 ^†^–29.67) (0.04–0.60)

^†^ <10^−2^ mW/kg.

**Table 5:** The distance d_lim_ (in m) for exposures generated by a single car as a function of the emitted power per antenna (33 dBm vs. 44.8 dBm) and the number of emitting antennas (one vs. four).

Human Model	33 dBm		44.8 dBm	
	One Antenna	Four Antennas	One Antenna	Four Antennas
Nina	1.5	3.5	8.5	20.5
Thelonious	4.5	9.5	21.5	77.5
Billie	6.5	10.5	40.5	43.5
Ella	8.5	13.5	19.5	50.5
Duke	5.5	12.5	24.5	46.5

**Table 6:** SAR_wb_ (in mW/kg) induced by two transmitting cars, where each car was positioned at a variable distance within a range of 13.5 m from the road user. Each transmitting car was equipped with either one (scenario C1) or four (scenario C2) emitting antennas, with each antenna operated at an emitted power level of 33 dBm. Details as in previous Table 3.

Human Model	d_i_ ≤ 13.5 m; Emitted Power Per Antenna: 33 dBm	
	Scenario C1: Two Cars, One Antenna	Scenario C2: Two Cars, Four Antennas
Nina	0 ^†^ (0 ^†^–0.15) (0 ^†^–0.01)	0.01 (0 ^†^–0.61) (0 ^†^–0.05)
Thelonious	0.04 (0 ^†^–1.77) (0.01–0.13)	0.16 (0 ^†^–7.08) (0.06–0.53)
Billie	0.04 (0 ^†^–1.59) (0.01–0.12)	0.14 (0 ^†^–6.37) (0.05–0.47)
Ella	0.03 (0 ^†^–1.20) (0.01–0.09)	0.11 (0 ^†^–4.79) (0.04–0.36)
Duke	0.03 (0 ^†^–1.19) (0.01–0.08)	0.11 (0 ^†^–4.74) (0.04–0.33)

^†^ <10^−2^ mW/kg.

**Table 7:** SAR_wb_ (in mW/kg) induced by three (scenarios D1, D3) and six transmitting cars (scenarios D2, D4), where each car was positioned at a variable distance within a range of 77.5 m from the road user. Each transmitting car was equipped with either one (scenarios D1, D2) or four (scenarios D3, D4) emitting antennas, with each antenna operated at an emitted power level of 44.8 dBm. Details as in previous Table 3.

Human Model	d_i_ ≤ 77.5 m; Emitted Power Per Antenna: 44.8 dBm			
	Scenario D1: Three Cars, One Antenna	Scenario D2: Six Cars, One Antenna	Scenario D3: Three Cars, Four Antennas	Scenario D4: Six Cars, Four Antennas
Nina	0 ^†^ (0 ^†^–0.98) (0 ^†^–0.02)	0.02 (0 ^†^–1.61) (0 ^†^–0.06)	0.01 (0 ^†^–3.92) (0 ^†^–0.08)	0.06 (0 ^†^–6.45) (0.02–0.26)
Thelonious	0.04 (0 ^†^–11.31) (0.01–0.21)	0.17 (0 ^†^–18.16) (0.05–0.71)	0.16 (0 ^†^–45.24) (0.04–0.86)	0.70 (0.02–72.63) (0.21–2.84)
Billie	0.04 (0 ^†^–10.34) (0.01–0.19)	0.16 (0.01–16.95) (0.05–0.63)	0.16 (0–41.35) (0.04–0.76)	0.65 (0.02–67.78) (0.21–2.52)
Ella	0.03 (0 ^†^–7.48) (0.01–0.16)	0.13 (0 ^†^–12.21) (0.04–0.49)	0.13 (0 ^†^–29.92) (0.04–0.62)	0.54 (0.02–48.82) (0.18–1.98)
Duke	0.03 (0 ^†^–7.18) (0.01–0.15)	0.13 (0 ^†^–11.36) (0.04–0.47)	0.13 (0 ^†^–28.72) (0.04–0.59)	0.51 (0.02–45.45) (0.17–1.90)

^†^ <10^−2^ mW/kg.

## Data Availability

Not applicable.

## Appendix A

### Appendix A.1. The Analytical Model of Field Propagation in V2V Communications

Below are the details of the deterministic model developed in [18] that we utilized in our study.

In [18], the LOS propagation condition was modelled with the two-ray ground reflection model [19]. According to this model, the incident electric field E_LOS_(d,t) (in V/m) at a generic distance d (in m) from the transmitting car and at time t (in s) can be calculated as follows [19]:

(A1) $$E_{\mathrm{LOS}}\left(d,t\right)=\frac{E_{0}d_{0}}{d_{1}}\cos\left(\omega_{c}\left(t-\frac{d_{1}}{c}\right)\right)+R_{\mathrm{ground}}\frac{E_{0}d_{0}}{d_{2}}\cos(\omega_{c}(t-\frac{d_{2}}{c})),$$

where E_LOS_(d,t) is measured on an horizontal plane, E_0_ (in V/m) is the free-space electric field at a reference distance d_0_ > d (in m), d_1_ (in m) is the distance travelled by the LOS component of the ray, d_2_ (in m) is the distance travelled by the ground-reflected ray, w_c_ (in rad/s) is the angular frequency of light, c (in m/s) is the speed of light, and R_ground_ is the reflection coefficient for ground.

The propagation under NLOS_v_ conditions (i.e., when the optical path between the transmitter and the receiver is obstructed by vehicles) was modelled in [18] with the multiple knife-edge diffraction model. In the presence of a single obstacle, the attenuation A_sk_ (in dB) of received power due to a single obstructing vehicle can be modeled using a single knife-edge model. The calculation of A_sk_ can be expressed as follows [20,29]:

(A2) $$A_{\mathrm{sk}}=\left\{\begin{array}{c} 6.9+20\log_{10}\left[\sqrt{{(v-0.1)}^{2}+1}+v+0.1\right];\;\mathrm{for}\;v\;>\;0.7\\ 0;\;\mathrm{otherwise} \end{array}\right.,$$

where $v=\surd 2\;H/r_{f}$, H (in m) is the difference between the height of the obstacle and the height of the straight line that connects the transmitting antenna with the receiver (i.e., the road user in our study), and r_f_ (in m) is the Fresnel ellipsoid radius. According to [20], in the presence of more than one obstacle, the attenuation can be modeled using a multiple knife-edge model by applying the ITU-R method described in [29].

The propagation under NLOS_b_ conditions (i.e., when the optical path between the transmitter and the receiver is obstructed by buildings) was modelled through the log-distance path loss model described in [21]. According to the log-distance path loss model, the path loss PL(d) (in dB) for a receiver at a distance d (in m) from the transmitting car can be calculated as follows [21]:

(A3) $$\mathrm{PL}\left(d\right)=\mathrm{PL}\left(d_{0}\right)+10{\gamma \log}_{10}\left(d/d_{0}\right),$$

where PL(d_0_) (in dB) is the path loss at a reference distance d_0_ > d, and g is the path loss exponent. The authors in [18] suggest to use g = 2.9, based on the dataset of measurements done in Porto downtown for the NLOS_b_ propagation condition. Finally, the resulting received power PR(d) (in dB) for a receiver at distance d is given by [18]:

(A4) $$\mathrm{PR}\left(d\right)=\mathrm{PT}-\mathrm{PL}\left(d\right),$$

where PT (in dB) is the power emitted by the transmitting car.

For the implementation of the deterministic model, we utilized the Matlab (The MathWorks, Inc., Natick, MA, USA) code developed by the authors of [18]. The code is available at: https://vehicle2x.net/ (accessed on 29 July 2023).

### Appendix A.2. The Stochastic Model to Account for the Variability of the Propagation Conditions in Real Urban Settings

Below are the analytical expressions of the probability functions developed in [22], which we utilized to simulate the variability of LOS, NLOS_v_, and NLOS_b_ propagation conditions in urban settings.

The probability PROB_LOS_(d) of LOS propagation condition at the distance d (m) from a transmitting car can be calculated as follows [22]:

(A5) $${\mathrm{PROB}}_{\mathrm{LOS}}\left(d\right)=0.8962e^{-0.017d}$$

Similarly, the probability PROB_NLOSV_(d) of NLOS_v_ propagation condition at the distance d (m) from a transmitting car can be calculated as follows [22]:

(A6) $${\mathrm{PROB}}_{\mathrm{NLOSV}}\left(d\right)=\frac{1}{0.0242d}e^{-\frac{{\left(\ln\left(d\right)-5.0115\right)}^{2}}{2.2092}}$$

The probability for NLOS_b_ propagation condition was derived by subtracting the sum of the probabilities of the LOS and NLOS_v_ propagation conditions from unity.
